# Will ^177^Lu-DOTATATE Treatment Become More Effective in Salvage Meningioma Patients, When Boosting Somatostatin Receptor Saturation? A Promising Case on Intra-arterial Administration

**DOI:** 10.1007/s00270-019-02262-1

**Published:** 2019-06-11

**Authors:** A. J. A. T. Braat, T. J. Snijders, T. Seute, E. P. A. Vonken

**Affiliations:** 1 Department of Radiology and Nuclear Medicine, University Medical Center Utrecht, Heidelberglaan 100, 3508 GA Utrecht, The Netherlands; 2 Department of Neurology, Brain Center Rudolf Magnus, University Medical Center Utrecht, Utrecht, The Netherlands

**Keywords:** PRRT, Lu-177-HA-DOTATATE, Meningioma, Neuro-oncology

## Abstract

Somatostatin receptor subtype 2 upregulation is very common in meningiomas, and the use of peptide receptor radionuclide therapy (PRRT) is recognized in recent European guidelines, with long-term stable disease and a long overall survival. Treatment efficacy of radionuclide treatments is correlated with tumour radiation absorbed dose. Meningioma patients with low tumour uptake might benefit less from treatment. Thus, a method to increase tumour uptake in these patients is needed. We describe a case treated with both intravenous and intra-arterial PRRT. Tumour uptake after intravenous PRRT was disappointing, and after intra-arterial administration significantly increased tumour uptake was seen. Patient had a partial response on imaging and reduction in tumour-related complaints. Potentially, intra-arterial administration of PRRT could increase treatment efficacy in meningioma patients.

*Level of Evidence* 5 (case report).

## Introduction

Somatostatin receptor subtype 2 (SSTR2) upregulation is very common in meningiomas. The potential of peptide receptor radionuclide therapy (PRRT) was recognized in recent European guidelines on meningioma [1]. The only currently available phase 2 prospective trial describing 34 patients showed long-term stable disease in 65.6% and a mean overall survival (OS) of 8.6 years. Looking at pre-treatment SSTR2 expression on imaging with indium-111-pentetreotide, the authors mentioned that on pre-treatment imaging, patients with high tumour uptake did better than those with low or intermediate uptake [4]. More recently, a case series study in 20 salvage meningioma patients found similar results [5]. Stable disease in 50% and median OS was not reached at a median follow-up period of 20 months. WHO I and II patients did better than WHO III patients, with stable disease after treatment in 100%, 57% and 13%, respectively. Notably, SSTR2 expression on gallium-68-DOTATOC PET imaging was significantly lower in WHO III meningiomas compared to WHO I/II meningiomas [5]. According to these two studies, treatment efficacy is related to SSTR2a expression. Logically, increased tumour uptake of the radiopharmaceutical results in a higher radiation absorbed dose. As expression of the somatostatin receptor in tumours cannot be increased, can we increase tumour uptake via other means and thereby increase treatment efficacy? Significantly increased uptake and objective response rates have been described in intra-arterial administration of PRRT in the hepatic artery of patient with neuroendocrine tumour as compared to intravenous administration [3]. We hypothesized that intra-arterial administration could be feasible in meningiomas as well, with increased efficacy compared to the intravenous route.

## Case Reports

**Fig. 1:**
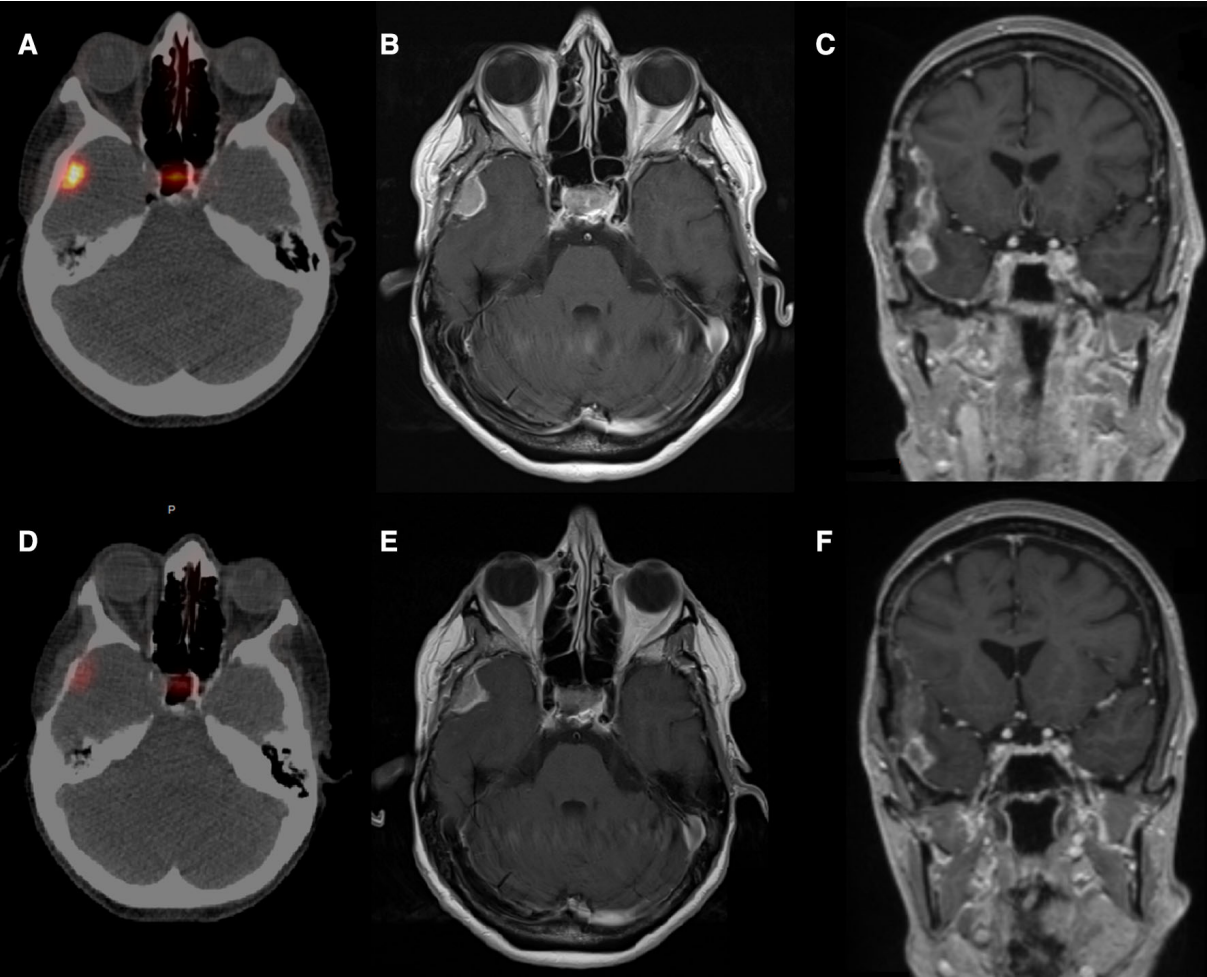
**A** Pre-treatment gallium-68-DOTATOC PET/CT showing high somatostatin receptor expression in the right temporal meningioma and physiological expression in the pituitary gland. **B **+ **C** Pre-treatment gadolinium enhanced T1 MRI sequence showing a homogeneously enhancing solid lesion. **D** Post-treatment gallium-68-DOTATOC PET/CT showed 79% decrease in SSTR2 expression. **E **+ **F** Gadolinium enhanced T1 MRI sequence showed a partial response of the right temporal meningioma, − 38% in volume and − 24% in longest diameter, and additionally central necrosis of the tumour can be acknowledged on the coronal reconstruction (**F**) and reduction in enhancement of tumour deposits in the old resection cavity (**F**)

**Fig. 2:**
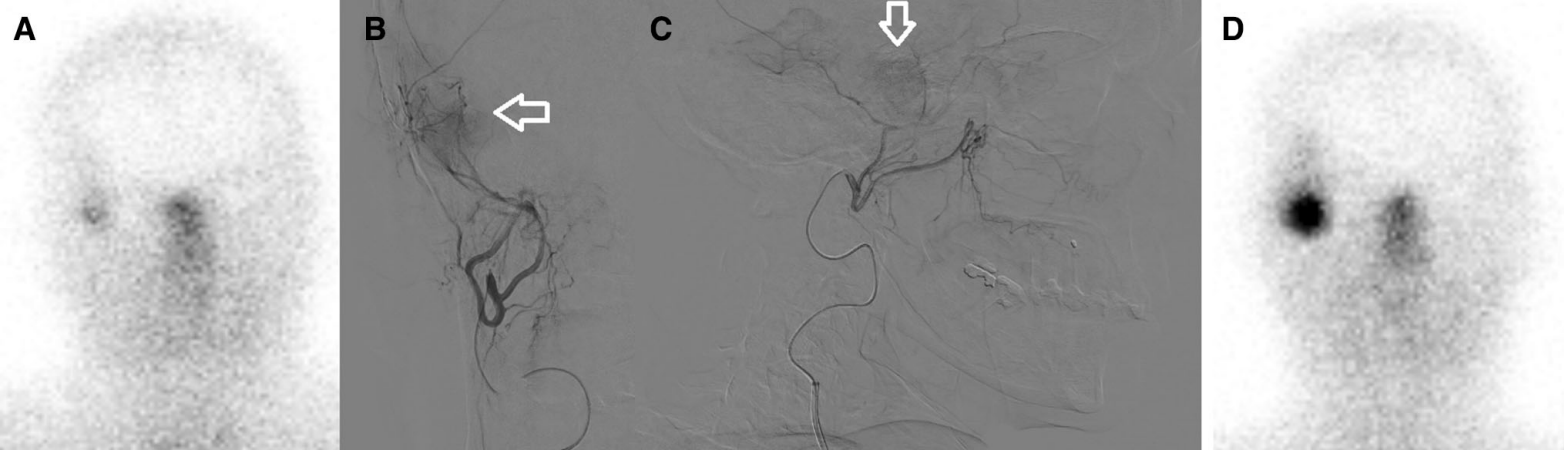
**A** Post-treatment lutetium-177-HA-DOTATATE scan after intravenous administration showing vague uptake of the radiopharmaceutical in the meningioma. **B **+ **C** Anterior and lateral digital subtraction angiography of the selective injection position in the right external carotid artery, middle meningeal artery, just proximal of the origin of the parotid artery. A tumour blush can be acknowledged (white arrows). Because an additional tumour feeding branch was originated from the parotid artery, this treatment position was chosen. **D** After intra-arterial administration, remarkable increase in radiopharmaceutical uptake is seen on the post-treatment lutetium-177-HA-DOTATATE scan and at quantification an 11-fold increase is measured

A 54-year-old female with a right temporal WHO II meningioma (Fig. 1A–C) was referred to our institute. She was refractory to conventional treatment with surgery (thrice) and radiotherapy (seven times). She suffered from focal seizures with sensory onset and impaired awareness 5x/day and every 2–3 months had a more severe focal seizure with impaired awareness, repeatedly leading to a status epilepticus. During multidisciplinary tumour board, the patients were considered irresectable by the neurosurgeon. The radiation oncologist had reached the maximum acceptable dose on surrounding healthy tissues; thus, additional external stereotactic radiation therapy was considered too risky. As meningioma is known for their SSTR2a overexpression, a ^68^Ga-DOTATOC PET/CT was performed to explore the option for PRRT (Fig. 1A). On imaging the tumour showed high SSTR2a expression, thus patients started with PRRT. She received 7.4 GBq ^177^Lu-HA-DOTATATE intravenously in February 2018 according to standard PRRT protocol for neuroendocrine tumours, with one night hospital admission according to local radiation safety regulations [6]. Post-treatment imaging obtained 24 h after the first cycle showed very low uptake in the tumour (Fig. 2A). Estimated absorbed dose was approximately 4,6 Gy (with the assumption of a residence time of 25.7 h) [2]. Four weeks after the treatment she had no clinical, biochemical or haematological toxicities. In stereotactic radiation therapy, a mean tumour absorbed dose of at least 50 Gy is pursued [2]; thus, intravenous PRRT was considered to result in a too low mean tumour dose. To increase tumour uptake of the radiopharmaceutical, we injected 7.4 GBq via a microcatheter placed selectively in the right external carotid artery via a femoral approach, after obtaining informed consent. Figure 2B shows the selective injection position in the right external carotid artery and middle meningeal artery. Thirty minutes prior to injection of the radiopharmaceutical, intravenous amino-acid infusion was started according to standard protocol for the duration of 4 h [6]. Via the microcatheter, the radiopharmaceutical was administered in 5 min with no additional side effects. On the post-treatment scan 24 h later, tumour uptake increased 11-fold (Fig. 2C), resulting in an estimated absorbed dose of 51 Gy. Four weeks after treatment, she experienced no side effects besides some local hair loss. We repeated the intra-arterial administration twice, aiming for a total > 150 Gy tumour absorbed dose. Two months after her fourth cycle (once intravenous and thrice intra-arterial, mean treatment interval 7 weeks; cumulative administered activity amounted to 29.6 GBq, estimated absorbed dose 154 Gy), her PET/CT showed significant decrease in SSTR2 expression and a partial response on MRI (Fig. 1D–F). Additionally, the evaluation MRI showed central necrosis of the meningioma and decreased diffusion restriction. Apart from limited localized hair loss after the second treatment, she experienced no treatment-related toxicities. More importantly, 3 months after her treatment regimen, her severe seizures have been absent since start of PRRT and her focal sensory-onset seizures decreased in frequency to 3–4x/week. Ten months after treatment, the frequency of the focal sensory-onset seizures was stable, her severe seizures were still absent, and the tumour remained in a partial response on MRI.

## Discussion

Based on this experience, intra-arterial PRRT might be a promising new treatment for meningioma patients’ refractory to conventional treatments. Similar to the initial findings on hepatic infusion of PRRT in neuroendocrine tumours, increasing the tumour absorbed dose via a selective intra-arterial injection may be possible. Additionally, as in this case, complaints/epileptic seizures caused by the tumour could be palliated effectively, increasing the patients’ quality of life. The short- and long-term toxicity profile of this treatment is expected to be similar as PRRT for neuroendocrine tumours (temporary bone marrow suppression, nausea and fatigue), as the radiopharmaceutical is a targeted agent [6]. Available literature on PRRT in meningioma is sparse, but promising. A combination of both stereotactic external radiation therapy and PRRT (combining high and low dose rate irradiation) seems to be safe and effective as well [2]. Future prospective studies on intra-arterial administration of PRRT (and potentially the combination with external radiation therapy) are needed to determine the safety and the long-term efficacy of this therapeutic modality in meningioma.

## Conclusion

Intra-arterial administration of PRRT might be a promising new treatment for meningioma patients’ refractory to conventional treatments.
